# Sensorimotor-linked reward modulates smooth pursuit eye movements in monkeys

**DOI:** 10.3389/fnins.2023.1297914

**Published:** 2024-01-09

**Authors:** Yongxiang Hu, Huan Wang, Mati Joshua, Yan Yang

**Affiliations:** ^1^Division of Life Sciences and Medicine, University of Science and Technology of China, Hefei, China; ^2^State Key Laboratory of Brain and Cognitive Science, Institute of Biophysics, Chinese Academy of Sciences, Beijing, China; ^3^Edmond and Lily Safra Center for Brain Sciences, The Hebrew University of Jerusalem, Jerusalem, Israel; ^4^Sino-Danish College, University of Chinese Academy of Sciences, Beijing, China

**Keywords:** reward, visual motion, smooth pursuit eye movements, initiation, steady state

## Abstract

Reward is essential for shaping behavior. Using sensory cues to imply forthcoming rewards, previous studies have demonstrated powerful effects of rewards on behavior. Nevertheless, the impact of reward on the sensorimotor transformation, particularly when reward is linked to behavior remains uncertain. In this study, we investigated how reward modulates smooth pursuit eye movements in monkeys. Three distinct associations between reward and eye movements were conducted in independent blocks. Results indicated that reward increased eye velocity during the steady-state pursuit, rather than during the initiation. The influence depended on the particular association between behavior and reward: a faster eye velocity was linked with reward. Neither rewarding slower eye movements nor randomizing rewards had a significant effect on behavior. The findings support the existence of distinct mechanisms involved in the initiation and steady-state phases of pursuit, and contribute to a deeper understanding of how reward interacts with these two periods of pursuit.

## 1 Introduction

Reward has powerful influences on motor behavior. In classic studies on animal reward processing, researchers have introduced animals with information regarding the type, quantity, and probability of rewards via proceeding sensory stimuli. These stimuli encompassed various factors, including the color (Bromberg-Martin and Hikosaka, 2011; Blanchard et al., 2015), shape (Watanabe and Sakagami, 2007; Matsumoto and Hikosaka, 2009), and location of visual targets (Takikawa et al., 2002; Summerside et al., 2018), the pattern of pictures (Ghazizadeh and Hikosaka, 2021), the auditory frequency (Coddington and Dudman, 2018), and other complex stimulus categorization (Pan et al., 2008). The results obtained from these tasks demonstrated that animals can make decisions based on the integration of indirect experiences, such as transitive inference (Mcgonigle and Chalmers, 1977; Bunsey and Eichenbaum, 1996), causal reasoning (Blaisdell et al., 2006; Hauser and Spaulding, 2006) and categorical inference (Herrnstein, 1979; Gardner and Lisberger, 2001). This enables them to select the appropriate cognitive response and guide their behavior through direct experiences of cue-guided rewards. Can reward information effectively drive behavior when it is exclusively associated with sensorimotor performances, in the absence of any additional sensory cues? In the hypothetical scenario where it was true, what is the inherent characteristic of the interaction between reward and the sensorimotor transformation?

The visually-guided smooth pursuit eye movements offer a valuable framework for comprehending these inquiries. The neural processing system involved in pursuit is responsible for computing the motion information of objects and identifying relevant salient information linked to a target via visual cues (Gardner and Lisberger, 2001) or other means. Previous studies using the classic step-ramp paradigm have shown that smooth pursuit eye movements consist of two distinct periods: the initiation, also referred to as the “open-loop” period, and the subsequent steady state, also known as the “closed-loop” period (Krauzlis and Lisberger, 1994). The pursuit initiation refers to the initial eye movement in reaction to target motion. This movement typically occurs within a duration of approximately 90–140 milliseconds after the initiation of pursuit (Lisberger and Westbrook, 1985). The latencies of pursuit initiation in human subjects exhibit a wide distribution ranging from 100 to 300 ms, as observed through the utilization of several visual targets in prior studies (Lindner and Ilg, 2000; Spering et al., 2005). The variation in eye responses during the initiation exhibits trial-to-trial fluctuations, and this motor variation can be attributed to sensory estimated errors in the neural system (Osborne et al., 2005). The initiation performance is greatly influenced by characteristics of visual objects, including shape (Tychsen and Lisberger, 1986), topological properties (Dou et al., 2023), the coherence of local motion (Behling and Lisberger, 2020), and textured backgrounds (Niemann and Hoffmann, 1997; Miura et al., 2009). In recent research, it has been reported that the eye velocity during the initiation can be substantially modulated by a preceding reward-related information, which is conveyed by the color of target (Joshua and Lisberger, 2012; Lixenberg and Joshua, 2018). The steady state commences subsequent to the end of pursuit initiation and remains constant during the whole pursuit process. During the period, eye movements are mostly guided by the feedback signals derived from motor performances. Behling and Lisberger recently found that the coherence within a patch of dots for a visual target can modify the smooth pursuit eye movement during both the initiation and steady state periods. The observed modulation effects indicated the existence of distinct neural mechanisms that regulate each of these two periods (Behling and Lisberger, 2020).

The primary aim of this study was to investigate the potential processing of the sensorimotor-linked reward signal within the brain system, as well as the manner in which reward interacts with the sensorimotor transformation to influence behavior. Within this context, there is an absence of a preceding sensory cue that serves as an indicator for the upcoming reward-related information. The reward settings were associated with eye velocity of smooth pursuit eye movements. Our study focused on eye responses during the pursuit initiation and steady-state tracking separately. Intriguingly, our results indicated that the modulation of sensorimotor-linked reward on pursuit eye movements is primarily observed during the steady state, as opposed to the pursuit initiation. The enhancement of eye responses can be achieved by exclusively rewarding greater eye velocity, as opposed to slower eye responses or utilizing a random order of reward. The findings depicted distinct reward interaction mechanisms during the sensory-driven initiation and the motor-supported steady-state of pursuit. Specifically, we observed that rewarding greater eye movements could serve as a condition for eliciting reward effects on the neural system.

## 2 Materials and methods

### 2.1 Animal preparation

In the experiments, three adult male rhesus monkeys (*Macaca mulatta*, 10–11 years old, 7.5–10 kg) were used. To prevent head motion during experimental sessions, monkeys were implanted with a head-holder using sterile technique under isoflurane anesthesia prior to experiments. In a second sterile surgical procedure, a coil was sutured to the sclera of one eye, enabling the recording of eye kinematics with high precision using the scleral coil technique. Following each surgical procedure, non-steroidal analgesics and antibiotic treatments were administered to monkeys for 3–5 days. After about 4 weeks of recovery, monkeys were trained to sit calmly in a suitable monkey chair (Crist Instruments, Bethesda, MD, United States) and pursue a moving target to get liquid reward. Before data collection in the study, all three monkeys had extensive experience conducting smooth pursuit eye movements to a moving target. They were previously trained to pursue a moving target at a constant velocity of 20 deg./s randomly in one of eight directions for a couple of months. Monkeys received an equal size of liquid as a reward for each pursuit trial. The animals were cared for in accordance with the Society for Neuroscience’s *Guide for the Care and Use of Laboratory Animals*.

### 2.2 Visual stimuli

An LED monitor (VG278Q, ASUS, China) with a 100 Hz refresh rate was positioned 40 cm from the monkey’s eyes and displayed visual stimuli. The horizontal and vertical spatial resolution of the monitor was 1,920 × 1,024 pixels, with a visual field of 74° × 46°. The background was a neutral gray (RGB: 128, 128, 128) with an average luminance of 19.19 cd/m^2^ (SM208 luminance meter, SANPOMETER, China). A 0.5° white square acted as both a fixation point and a visual stimulus target for pursuit.

Trials started with an initial fixation point located in the center of the screen for a randomly selected period between 600 and 1,000 ms to minimize anticipatory movements, and monkeys were required to fixate within an invisible 2° × 2° window. The fixation square served as a target for step-ramp motions following effective fixation (Rashbass, 1961). It shifted 3° eccentrically and moved leftward or rightward at 20°/s in distinct blocks. The target motion continued for 820 ms before the trial terminated with a 300 ms fixation on the stationary spot. Monkeys maintained their tracking eyes within a 4° × 4° invisible window surrounding the target to complete the entire trial. At the end of trials, fluid drops might be delivered as rewards based on various reward block designs.

### 2.3 Behavioral tasks

**Figure 1:**
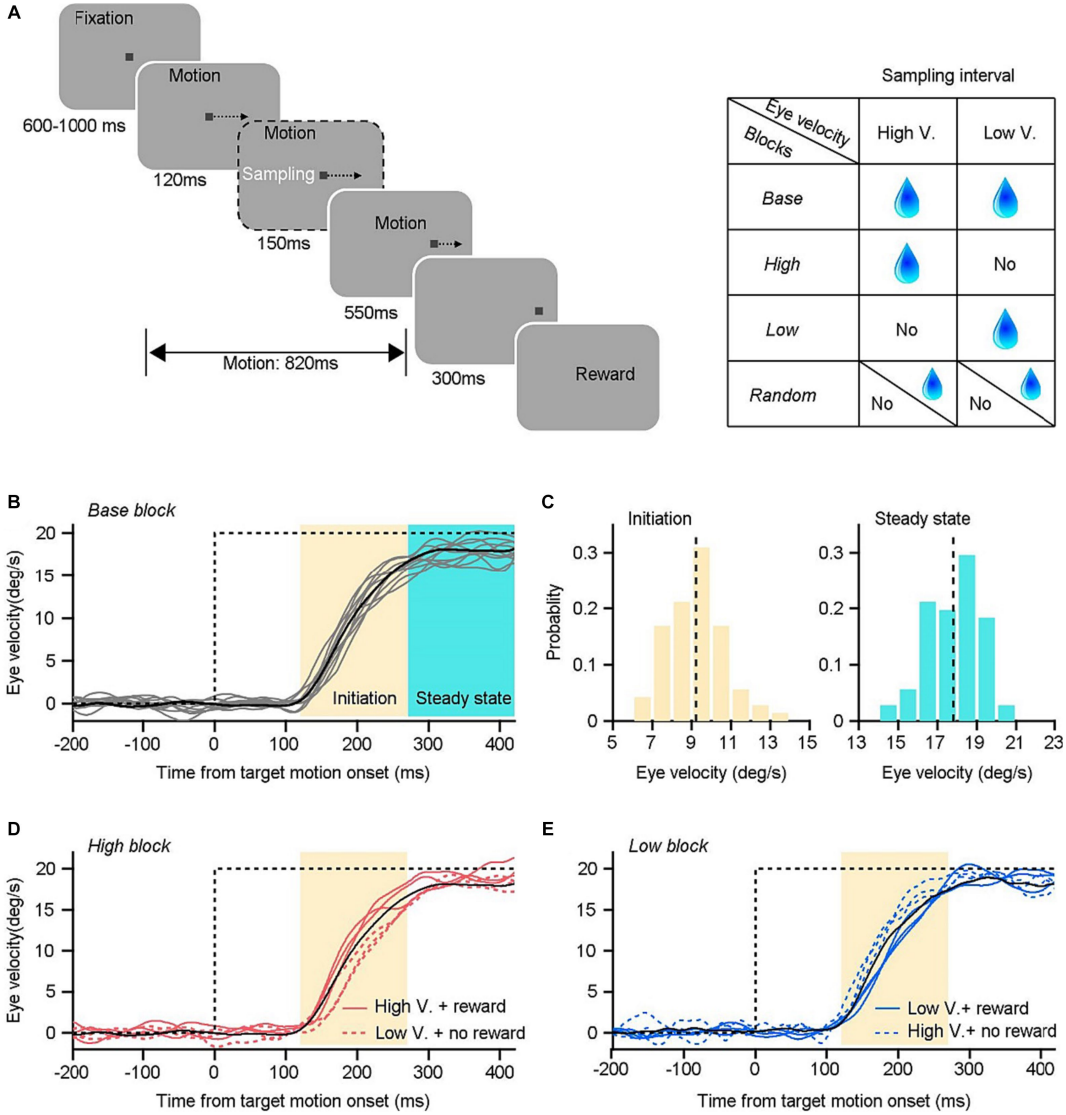
Experimental design and example trials in an example experimental day. **(A)** Visual stimulation design and sensorimotor-linked reward settings. **(B)** Horizontal eye velocity as a function of time in the base blocks. Black line is the mean eye velocity trace averaged across individual trials (gray lines). Time interval from 120–270 ms (yellow area) after the onset of target motion is the initiation period of pursuit, and 270–420 ms (cyan area) is the time interval of steady-state pursuit. **(C)** Distribution of eye velocity during the initiation (left) and steady state (right) of pursuit eye movements. **(D)** Eye velocity traces in a high block. Monkeys only received rewards within trials with higher eye velocity during the initiation period (red solid lines) than the threshold in the associated base block (the black line). **(E)** Eye velocity traces in the low block. Monkeys only received reward in trials with lower eye velocity during the initiation period (blue solid lines) than that in the associated base block (the black line).

In the study, whether monkeys could receive liquid drops as a reward on a given trial or not depended on their sensorimotor performances on individual trials and distinct reward setting rules of that block. A customized microcontroller unit (STM32F103C8T6, STMicroelectronics, NV) was utilized to collect and calculate monkeys’ horizontal eye trace signals during the sampling interval on each trial. The data was collected at a rate of 1 kHz. Only the eye movements within the sampling interval were used to determine whether the reward should be delivered on that trial. In the main experiment, the sampling interval was a time window of 150 ms, starting from 120 to 270 ms after the onset of target motion (initiation interval, yellow shading in Figure 1B). In the control experiment, the sampling interval was shortened into 100 ms, from 120 to 220 ms. The control experiment was conducted in separated blocks on different experimental days.

The behavioral tasks consisted of four distinct blocks that involved reward settings. In each block, the reward delivery was linked to animals’ behavioral performances according to specific rules (Figure 1A). In *base blocks*, monkeys were provided with a reward of 0.2 mL fluid drops in each successfully completed trial. Each base block consisted of 100 trials. Monkeys’ eye velocity during the sampling interval can be obtained by a customized microcontroller unit, enabling real-time calculations. The distribution of eye velocities within the sampling interval and the average eye velocity across these 100 trials were analyzed once monkeys finished the base block. The average value was stored by the microcontroller unit and served as a threshold for the subsequent behavioral blocks. The reward rules in the other three blocks were designed according to the relationship between the eye movement on individual trials and the threshold. The eye velocity during the sampling interval in each trial was measured and compared to the threshold value by the microcontroller unit. In *high blocks*, monkeys were provided with a fluid reward of 0.2 mL in a given trial, but only if their eye velocity in that trial exceeded the threshold determined from the base block. In the event that the eye velocity fell below the designed threshold, the delivery of reward would be withheld. In *low blocks*, an opposite reward rule was utilized. Only when the sampled eye velocity in a given trial was lower than the threshold, monkeys could get a 0.2 mL reward. In *random blocks*, monkeys received 0.2 mL fluid with a random probability of 50% determined by chance, regardless of their eye movements. Each of the three blocks contained 200 trials.

In each experimental day, the base blocks were randomly followed by one of the other three reward setting blocks. Consequently, the threshold for each high, low and random blocks depended on their prior base blocks. Even monkeys successfully track the moving target, they will have a chance that trials yield no reward according to distinct reward setting in that block. All three monkeys are well trained for pursuing target motion. They attempted about 1,500 to 2,000 trials in the experiments and their completion rates were above 92% (monkey A, 98%; C, 92%; L, 98%).

### 2.4 Data acquisition and analysis

We measured eye position signals from a magnetic search coil system (Crist Instruments, Bethesda, MD, United States) in order to estimate horizontal and vertical eye movements. Voltages proportional to horizontal and vertical eye velocity were generated after passing the signals through an analog differentiator. The differentiator contained a filter that rejected signals above 25 Hz (−20 dB per decade) and produced eye movement signals at lower frequencies. Each channel’s ocular signals were sampled at 1 kHz and stored for off-line analysis.

All analyses were performed in MATLAB (MathWorks, United States). Each trial’s ocular traces were inspected by a custom-built MATLAB program. According to an eye acceleration threshold of 400 deg./s^2^, saccadic eye movements were identified during an analysis interval between 120 and 420 ms after the onset of target motion. Then, we manually rechecked each trial to account for any omissions or false detections. Trials were discarded if saccade occurred within the interval.

The initiation of pursuit in individual trials is detected automatically. Initially, the pursuit latency was established according to the mean eye velocity measured within each block (Carl and Gellman, 1987; Lindner and Ilg, 2000). A 150 ms analysis window, spanned from 100 ms before to 50 ms after the onset of target motion, was utilized to calculate the baseline (average) of the mean eye velocity during fixation. If the mean eye velocity deviated from the baseline by more than three times its standard deviation of eye velocity within the next 50 milliseconds, a linear regression analysis was performed on the eye velocity data during that time period. The intersection time point of the regression line and the baseline was defined as the mean pursuit latency in that block. We further estimated the onset time of pursuit in single trials utilizing the mean pursuit latency, as proposed by an established method (Lee and Lisberger, 2013; Dou et al., 2023). The mean eye velocity throughout a 120 ms interval, ranging from 20 ms before to 100 ms after the pursuit initiation, is set as a template. In order to obtain the best least-squares fit to the eye velocity in each trial, the template was adjusted by shifting on the time scale and scaling on the eye velocity scale. During the fitting process, the free parameters of time shift and velocity scaling were provided. The pursuit latency of a single trial was computed by utilizing the shifted time value that provided the greatest fit.

Unless otherwise specified, behavioral data were evaluated using a paired Student’s t-test with two-tailed hypothesis testing. With a significance level of 0.05, statistical analysis was conducted by assessing for significance across experimental blocks.

## 3 Results

Our study employed visually-guided smooth pursuit eye movement tasks to probe how reward interacts with sensorimotor transformation in the absence of additional preceding sensory cues for the reward. First, a novel reward setting was devised wherein the delivery of reward in a given trial was associated with the eye velocity in that trial. Second, we demonstrate that the reward associated with sensorimotor performances exerts distinct effects on both the initiation and steady state of pursuit. Only associating reward with greater eye velocity during the pursuit initiation led to faster eye movements during the pursuit steady state. The effect of reward did not impact eye responses if reward was associated with slower pursuit eye movements or delivered in a randomized order. Third, we have demonstrated that the association between the eye velocity and reward was crucial. Neither motor experience nor the presence of reward in the prior trial modulated eye velocities effectively.

### 3.1 Reward delivery linked with sensorimotor performances in pursuit

Smooth pursuit eye movement is the natural tracking behavior that can be elicited by the motion of a small target across the visual field. In response to repetitive presentations of the identical target motion in the step-ramp paradigm, the pursuit response exhibits variable latencies ranging from 80 to 125 ms after the onset of motion in monkeys (Krauzlis and Lisberger, 1994; Krauzlis and Miles, 1996; Lee et al., 2016). The behavior is illustrated in Figure 1, wherein the target underwent a rightward motion at 20°/s. Figure 1B depicts the variation in horizontal eye movements in response to the identical target motion within the first 420 ms following the onset of target motion. Each gray line represents the ocular responses on single trials conducted on an example experimental day. It indicates that the level of pursuit performances varies across multiple trials and over time within each individual trial.

We analyzed the horizontal components with a 1-ms resolution throughout two intervals of 150-ms (Figure 1B), starting from 120 to 270 ms or 270 to 420 ms after the onset of target motion. Both analysis intervals emphasize on the initiation and steady-state of pursuit, respectively. The initiation interval is the part of the response that is driven by the initial visual motion before there has been modulated by visual feedback. The steady-state tracking is driven largely by the feedback of motor commands instead of retinal image motion. Because of the different mechanisms underlying initiation and steady state periods of pursuit, we study these two analysis intervals separately. The sampling interval from 120 to 270 ms may go slightly beyond the end of the open-loop interval in some data sets. However, none of our results or conclusions depended strongly on whether we made our measurements within a window of 100, or 150 ms after the initiation of pursuit (Mann–Whitney-Wilcoxon test, *n*_100_ = 13, *n*_150_ = 21, *p* = 0.5950).

To enhance comprehension of the processing of reward signals in guiding sensory-motor transformations, we devised specific associations between sensorimotor parameters within the sample interval and rewards in various setting block (Figure 1A). The base block provides natural properties of smooth pursuit eye movements to a moving target, including mean eye velocity and its variance (Figure 1C). In our majority experiments, we measured the mean response within the initiation interval of 150 ms, which serve as a threshold for the sensorimotor-linked reward setting. In the high block, monkeys were provided a fluid reward in a specific trial if their eye velocity during the sampling interval in that trial (shown by solid red lines in Figure 1D) exceeded the threshold determined from the base block (the black line in Figure 1D). In the low block, there exists an inverse association between reward and eye responses. Monkeys were only rewarded when their eye velocity (shown by solid cyan lines in Figure 1E) fell below the predetermined threshold (the black line in Figure 1E). Additionally, we incorporate the random block as a means of control. Monkeys were administered fluid with a probability of 50% that was chosen randomly, independent of their eye responses. In contrast to cue-guided reward behavioral paradigms, the proposed sensorimotor-linked reward design offers a direct method to probe the effects of reward which is associated with subjects’ behavioral performances.

### 3.2 Effects of sensorimotor-linked reward on pursuit initiation and steady state

**Figure 2:**
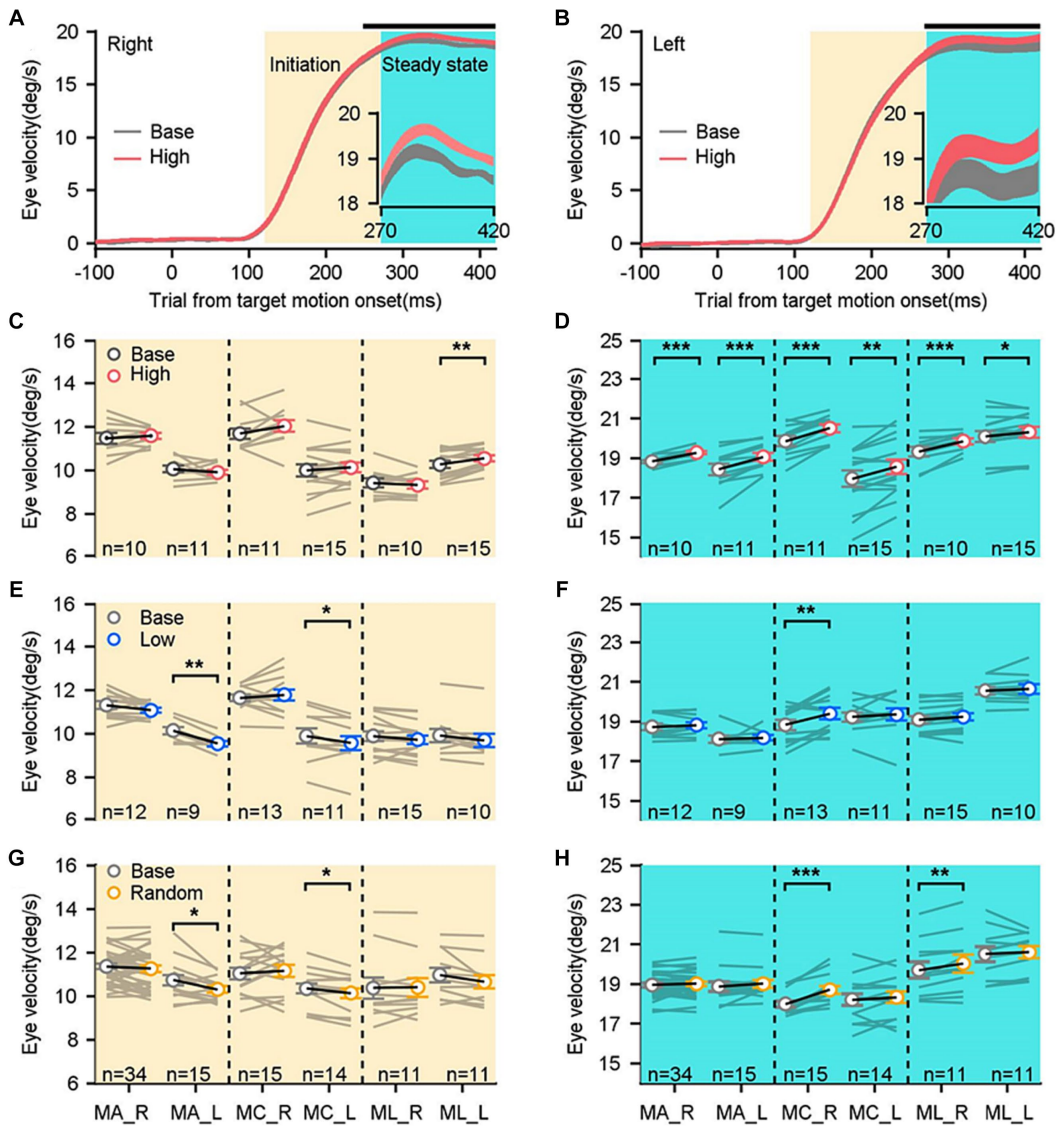
Comparisons of smooth pursuit eye movements in distinct sensorimotor-linked reward settings. **(A,B)** The average eye velocities across all base blocks (gray trace) compared to those across all high blocks (red trace) along the rightward and leftward directions in an example monkey. The black dots shown on the top of the graph indicated the time points at which significant differences of eye velocities were observed. The insets offer a magnified representation of the eye responses during the period. **(C,E,G)** Comparisons show the eye velocities in the high block **(C)**, the low block **(E)**, the random block **(G)**, and their associated base blocks during the initiation of pursuit. **(D,F,H)** Comparisons of eye velocities during the steady state of pursuit. Gray lines show data in a given reward setting block and its associated base block. Each circle and black line show mean data across blocks. Error bars: Mean ± SEM. ^*^p < 0.05; ^**^p < 0.01; ^***^p < 0.001, Paired Student’s *t*-test.

To examine whether and how the sensorimotor-linked reward affects behavioral performances, we compared eye responses of three monkeys during the initiation and steady state under three distinct reward settings. An example of monkey’s eye velocity traces to the rightward and leftward target motion were illustrated in Figures 2A,B. We computed the average eye velocity in the base block (gray lines) and the high block (red lines) across all experimental blocks. The sensorimotor-linked reward to either target motion direction did not significantly alter eye velocity during the initiation interval (gray shading; Figure 2A, *t*(9) = −0.61, *p* = 0.5547; Figure 2B, *t*(10) = 0.96, *p* = 0.3593). While, the eye responses in the steady state interval showed significant increases associated with rewarding greater eye velocities during the pursuit initiation in both rightward (cyan shading and subplots, Figure 2A, *t*(9) = −8.29, *p* < 0.0001) and leftward (Figure 2B, *t*(10) = −5.01, *p* = 0.0005) directions. These results were consistent among three studied monkeys. Reward greater eye velocities during the initiation interval failed to cause significant changes in pursuit initiation (Figure 2C, *n* ≥ 10 experimental blocks, *p* > 0.15), whereas only increased eye velocities in monkey L when he tracked leftward motion (*t*(14) = 3.68, *p* = 0.0025). This reward setting significantly facilitated eye movements during the steady state interval of pursuit in both horizontal directions for all three monkeys tested (Figure 2D, *n* ≥ 10 experimental blocks, *p* < 0.05).

In the low block, we inverted the association between reward and eye velocity. Monkeys were rewarded when their eye responses during the initiation interval were below the threshold. Intriguingly, there were no consistent changes in the eye velocities neither during the pursuit initiation (Figure 2E, *n* ≥ 9 experimental blocks) nor during the steady state interval of pursuit (Figure 2F) for all three monkeys in both horizontal motion directions. Then we tested the effects in the random block, where the fluid reward was delivered randomly with a probability of 50%, regardless of monkeys’ ocular responses (Figures 2G,H). Across the three monkeys and two motion directions, there were no consistent changes of eye responses in the initiation of pursuit (Figure 2G, *n* ≥ 11 experimental blocks) and the steady state of pursuit (Figure 2H). Consequently, only the association of reward with greater eye velocity during the initiation interval facilitated consistently pursuit eye movements during the steady state.

**Figure 3:**
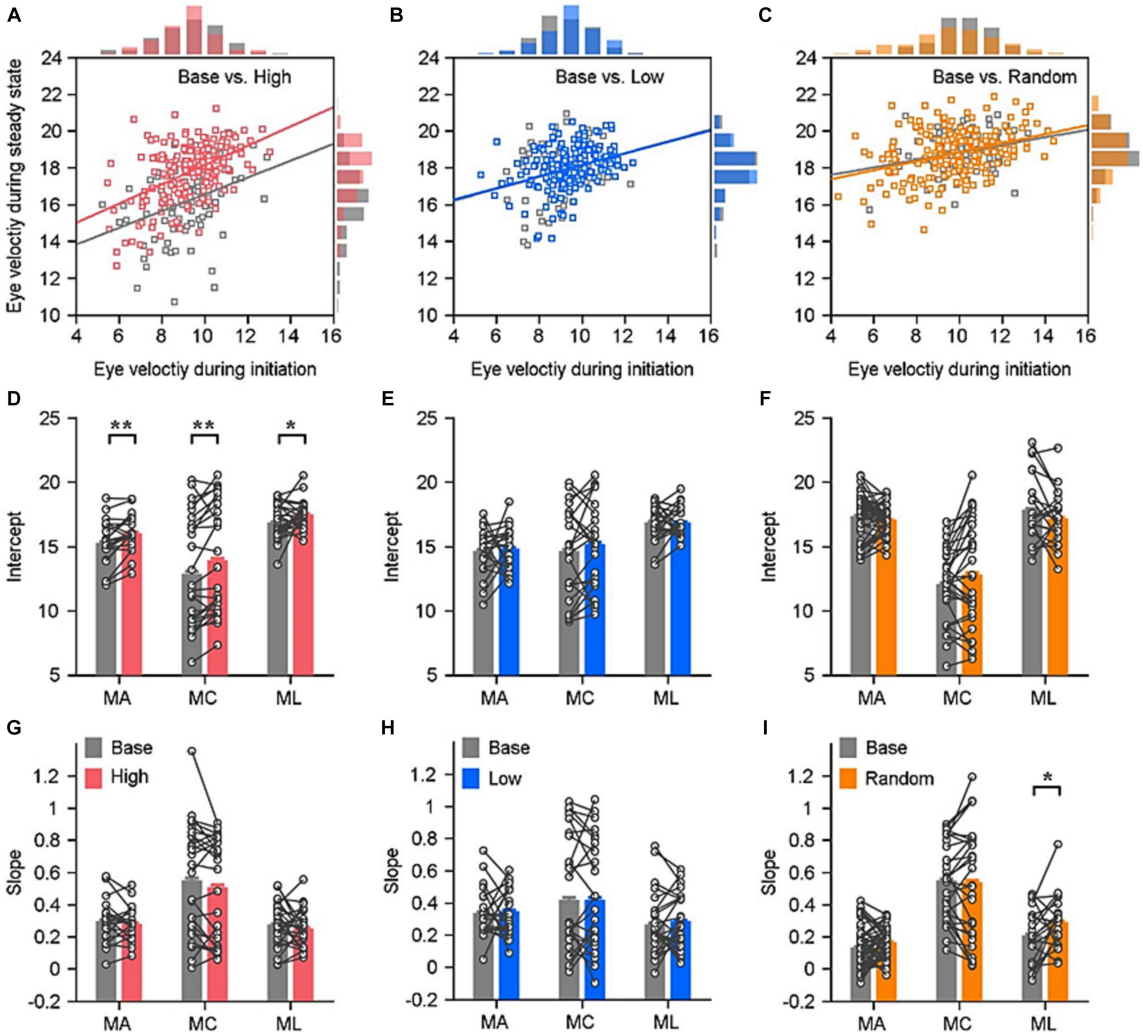
Quantitative assessment of relationships between ocular responses in the steady state and the initiation in various reward settings. **(A–C)** Demonstration of how the eye velocities during the steady state of pursuit depending on the eye velocity during the pursuit initiation in an experimental day of the example monkey. Solid lines are linear fits for data in different reward settings (color lines) and their base block (gray lines). The distribution of eye velocities is displayed in the histograms. Each circle represents the data of individual trial in that block. **(D–I)** Quantitative evaluation of ocular responses’ linear fitting in the three reward settings: the intercept **(D–F)** and the slope **(G–I)**. Each circle and line show data in a given reward setting block and its associated base block. Error bars: Mean ± SEM. ^*^p < 0.05; ^**^p < 0.01; Paired Student’s *t*-test.

The effect of sensorimotor-linked reward on eye movements varied quantitatively depending on the reward settings. Previous research indicated that the pursuit initiation varies from trial to trial, and that this variation is correlated across time within individual trials. According to our data, eye responses that evoke pursuit with slower or faster eye velocities in the initiation interval tend to maintain slower or faster eye velocities during the steady-state tracking. We plotted the correlation between eye responses during the two periods, the eye velocity for pursuit steady state as a function of its eye response for pursuit initiation in individual trials (Example blocks in Figures 3A–C). To mathematically evaluate these differences, we performed a linear regression analysis on eye velocities when monkey tracked leftward or rightward motion associated with distinct reward settings. Individually, we computed slope and intercept of the fitted line for each block. The intercepts of eye responses in the high block were significantly greater than those in the base block for all three monkeys (Figure 3D, Monkey A, *t*(20) = −3.57, *p* = 0.0019, Monkey C, *t*(25) = −3.34, *p* = 0.0026, Monkey L, *t*(24) = −2.07, *p* = 0.0492). While, there were no significant differences between the slopes of eye response correlations in the base and high blocks (Figure 3G, monkey A, *t*(20) = 0.82, *p* = 0.4177, monkey C, *t*(23) = 1.47, *p* = 0.1533, monkey L, *t*(24) = 0.77, *p* = 0.4496). When the reward was associated with lower eye velocity, there were no significant differences between the intercepts compared to their associated base blocks (Figure 3E, monkey A, *t*(20) = −0.36, *p* = 0.7215; monkey C, *t*(23) = −1.05, *p* = 0.3004; monkey L, *t*(24) =0.04, *p* = 0.9656). There were no significant alterations in the intercepts when the reward was delivered randomly (Figure 3F, monkey A, *t*(48) = 1.08, *p* = 0.2875; monkey C, *t*(28) = −1.54, *p* = 0.1348; monkey L, *t*(21) = 1.48, *p* = 0.1522). Neither in the low block nor in the random block can the reward significantly modulate the slope of correlation of eye velocities across the three tested monkeys (Figure 3H, *p* > 0.5 for all three monkeys; Figure 3I, *p* > 0.1 for monkey A and C, *p* = 0.036 for monkey L).

We have conducted a parallel analysis to evaluate whether the facilitation on pursuit is related to the change of reward ratio in the high block. We first assessed if the reward ratio varied between the high block and its corresponding base block. In the base block, we calculated the probability of trials with greater or lower eye velocities than the threshold. This probability could serve as a “sham” reward ratio that monkeys might receive in that block if the setting of rewarding greater eye velocity was to be applied. There are no significant changes in the reward ratio between these two blocks (*n* ≥ 10, *p* > 0.05). Furthermore, there was no significant correlation between the reward ratio and the facilitation on pursuit movement in the steady state of the high block (Pearson’s correlation coefficient, *r* = 0.06, *p* = 0.6730). Consequently, the reward ratio could not account for the increase in pursuit eye velocity in the high block.

In our data analysis so far, trials containing saccadic eye movements during the analysis intervals were discarded. To further verify whether the distinct sensorimotor-linked reward settings modify pursuit responses by increasing or decreasing catch-up saccadic eye movements, the probability of saccade trials within individual blocks was calculated. A comparison of the saccade-trial probability in the base block revealed that monkeys improve their tracking with fewer saccadic eye movements in all high and low blocks (*n* ≥ 70, *p* < 0.001). However, these probabilities of saccade did not differ significantly between high and low blocks (unpaired two-tailed Student’s *t*-test, *n*1 = 70, *n*2 = 72, *p* > 0.05). Instead of influence on catch-up saccadic eye movements, the observed facilitation of eye movements by rewarding greater ocular responses could be modified in the pursuit system.

**Figure 4:**
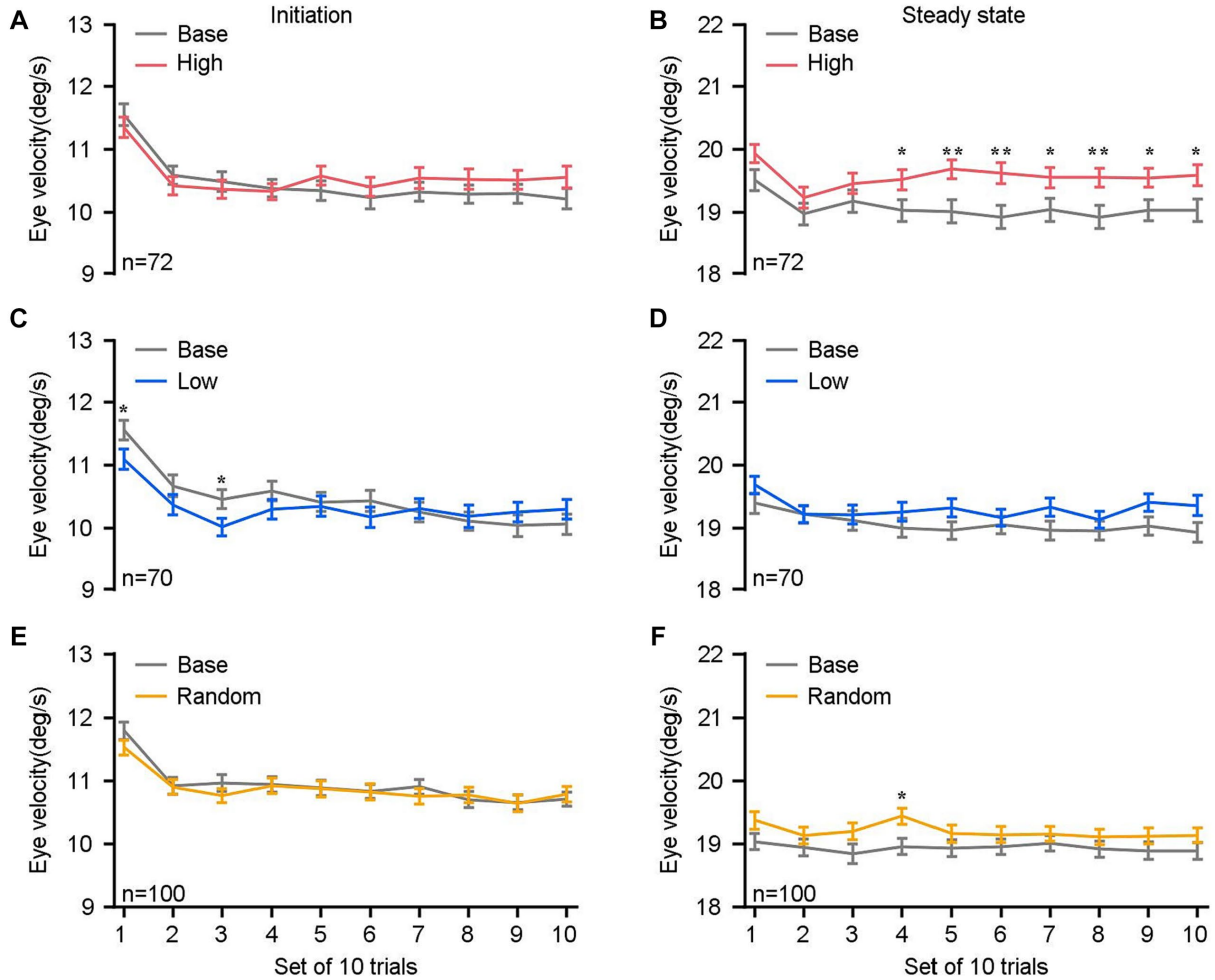
Comparisons of eye velocities as a function of sets of 10 trials. **(A,C,E)** The mean eye velocity within a 150 ms interval of the initiation of pursuit, from 120 to 270 ms after target motion. **(B,D,F)** The mean eye velocity within a 150 ms of the steady-state pursuit, from 270–420 ms after target motion. The eye velocities in the high block (red lines in **A,B**), the low block (blue lines in **C,D**), the random block (yellow lines in **E,F**), and their respective base blocks (gray lines) are compared. The *n* denotes the total number of blocks across experimental days and monkeys within each specific reward setting. Error bars: Mean ± SEM. ^*^p < 0.05; ^**^p < 0.01.

To understand what was really happening during monkeys experienced different sensorimotor-linked reward settings, we further conducted an analysis of eye motion in relation to trial sequences. The eye velocities during the initiation of pursuit (Figures 4A,C,E) as well as the steady-state phase (Figures 4B,D,F) were evaluated as a function of sets of 10 trials across all blocks and animals. To facilitate comparison with the base block, our analysis was specifically directed at the first 100 trials within each block. It was observed that the eye velocities during the steady-state phase of pursuit in high blocks exhibited a statistically significant increase compared to the corresponding base blocks (Figure 4B). The sustained efficacy of eye movement enhancement has been observed following the first thirty trials (*n* ≥ 70, *p* < 0.05). Again, this difference was not observed during the initiation phase (Figure 4A). The findings presented here indicate that the impact of sensorimotor-linked reward on pursuit performance may necessitate a substantial number of trials involving sensorimotor experiences in order to be successful, particularly in high blocks.

In light of these findings, sensorimotor-linked rewards indeed modulate smooth pursuit eye movements effectively, without the need for additional sensory cues for the upcoming reward. Among distinct associations between the sensorimotor parameters and reward, only associating reward with greater eye responses could facilitate pursuit eye movements. Associating reward with slower eye movements or at a random chance did not significantly modify behavioral performances. In addition, this facilitation only works for the motor-feedback supported steady-state of pursuit, as opposed to the sensory-estimation driven initiation of pursuit.

### 3.3 Pursuit latency remains consistent under distinct reward settings

Previous studies in nonhuman primates have shown that reward associated with sensory cues could evoke saccadic eye movements with shorter latencies (Watanabe et al., 2003) and larger peak eye velocities (Takikawa et al., 2002) compared with no reward condition. In mice, the anticipation of reward can facilitate their operant behavior, resulting in a shorter movement latency (Ishino et al., 2023). By contrast, the threat of punishment strengthens the constraints on operant behavior to obtain reward, resulting in longer movement latency (Verharen et al., 2019). In smooth pursuit eye movements, pursuit latency and gain of pursuit were positively correlated, indicating that monkeys would move faster when the initiation was late (Osborne et al., 2005). Thus, the enhancements of eye movements during the steady-state phase might be caused by changes of the initiation of pursuit.

**Figure 5:**
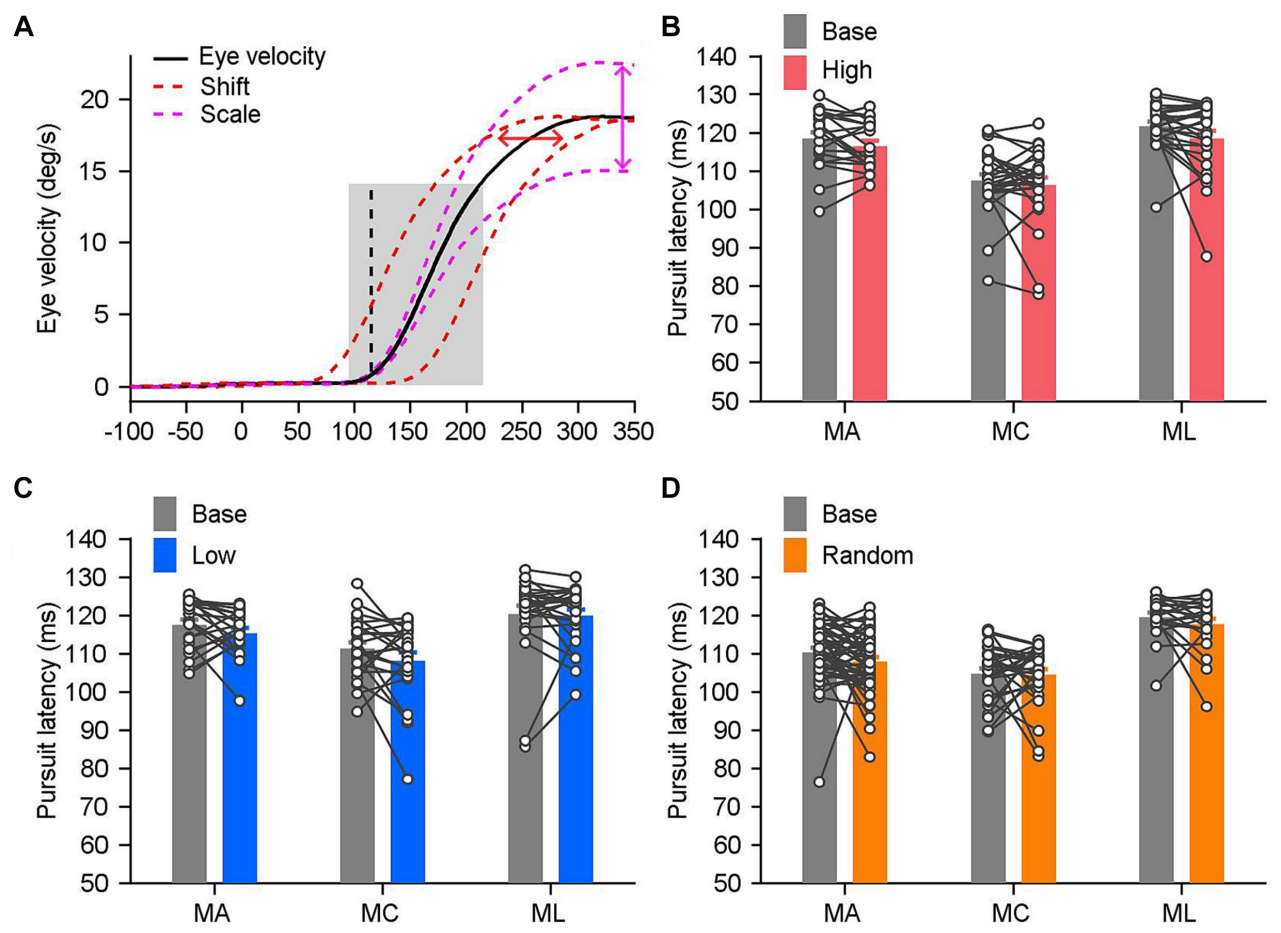
The onset time of pursuit was consistent under distinct reward settings. **(A)** The method utilized to estimate the latency of pursuit in single trials. The mean eye velocity (black solid trace) within the gray shading area, ranging from 20 ms before to 100 ms after the pursuit initiation (black dashed line), is set as the template. In order to obtain the best least-squares fit to the eye trace of single trial, the template was adjusted by shifting on the time scale (the x axis, red dashed traces) and scaling on the eye velocity scale (the y axis, magenta dashed traces). **(B–D)** The mean latency of pursuit in the individual block under distinct reward settings: the high block **(B)**, MA: *n* = 21, MC: *n* = 26, ML: *n* = 25; the low block **(C)**, MA: *n* = 21, MC: *n* = 24, ML: *n* = 25; and the random block **(D)**, MA: *n* = 49, MC: *n* = 29, ML: *n* = 22. Each line and the paired circles present data in a given reward setting block and its associated base block. Error bars: Mean ± SEM.

We examined whether the latency of pursuit changed under the various sensorimotor-linked reward settings to test this hypothesis. Three monkeys were used to assess the pursuit latency of each individual block, which ranged between 70 and 125 ms (Figure 5A). Between the base blocks and the high blocks, there were no significant differences in the pursuit latencies among the three monkeys (Figure 5B; *n* ≥ 21, *p* > 0.05 for three monkeys). Between the base blocks and the low or random blocks, we were also unable to detect any significant variations in the pursuit latencies (Figure 5C, base blocks vs. low blocks, *n* ≥ 21, *p* > 0.12 for three monkeys; Figure 5D, base blocks vs. random blocks, *n* ≥ 22, *p* > 0.1 for three monkeys). These findings suggested that the initiation of pursuit eye movements was unaffected by sensorimotor-linked reward.

We also examined how pursuit latency varied across various reward settings. The variation of the onset time of pursuit served as a sensitive behavioral measure to probe the process of sensorimotor transformation. It might be influenced by a variety of factors, including visual inputs, motivation, and rewards. We calculated the variation in pursuit latency across individual trial in each block and compared it with the associated base block. The variance in pursuit latency was unaffected by the setting of sensorimotor-linked reward (base vs. high, and base vs. low for three monkeys, *n* ≥ 70, *p* > 0.3). Thus, the enhancement of smooth pursuit eye movements was not caused by any alterations of the initiation of pursuit. Greater eye movements that are rewarded do in fact facilitate the eye responses during the steady state of pursuit.

### 3.4 Effects of prior trial’s motor and reward experiences on pursuit

In our study, sensorimotor-linked rewards enhanced eye velocities during the steady-state of pursuit when liquid rewards were associated with faster eye movements during the initiation of pursuit. The occurrence of regression towards the mean is a widely recognized phenomenon in the domain of behavioral performances. This phenomenon is characterized by a tendency for faster eye velocities in the preceding trial to be followed by slower eye velocities in the subsequent trial, and vice versa. An unanticipated reward may evoke perceptional attention in order to facilitate a more efficient sensorimotor transformation, thereby facilitating behavior generation. Thus, the enhancements observed in our results may be a result of the behavioral performance and the occurrence of reward in the prior trial, or the rule of association between sensorimotor responses and reward across the entire block. Two additional analyses were conducted to verify these hypotheses.

**Figure 6:**
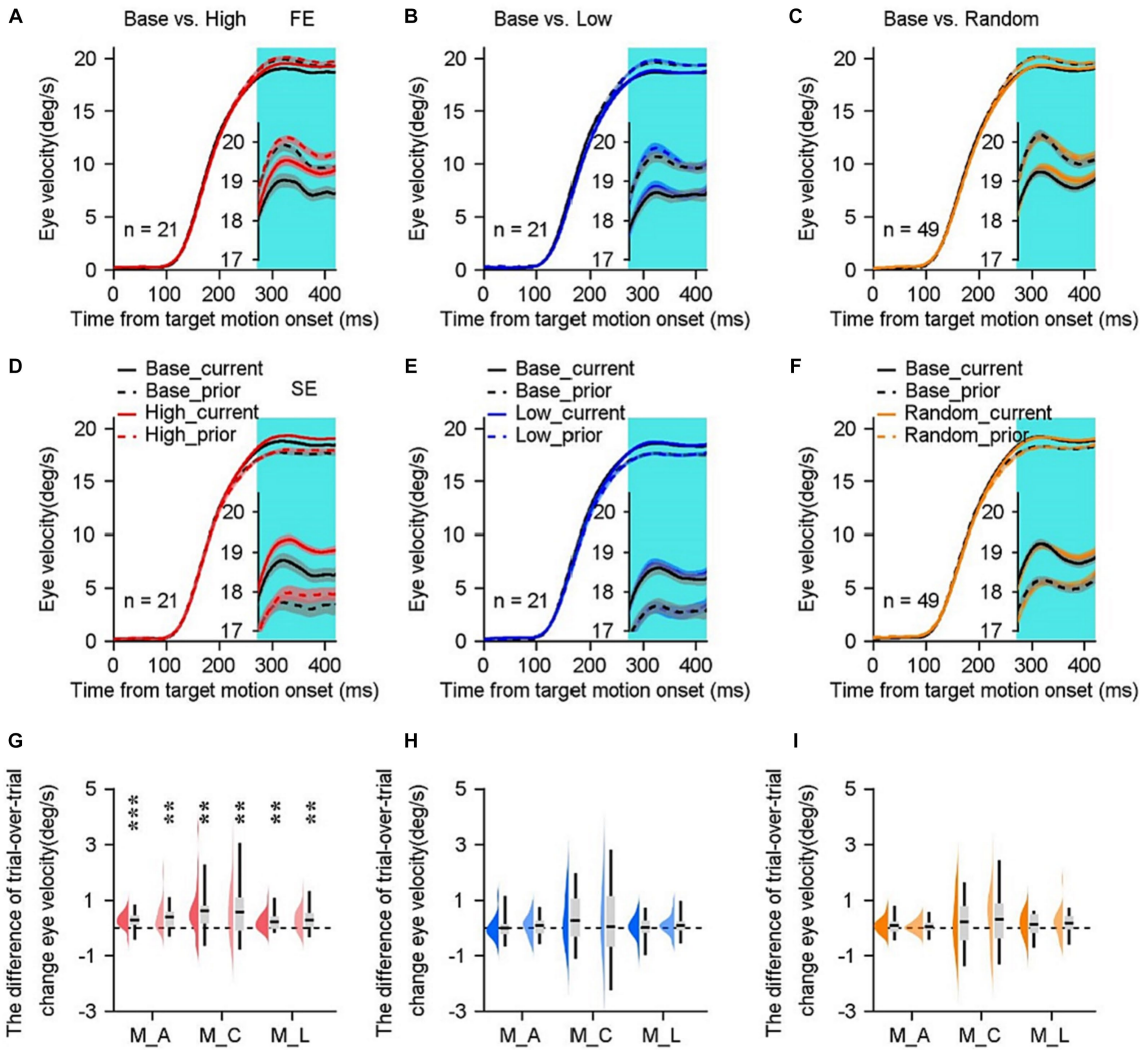
Trial-over-trial eye velocity changes vary in distinct sensorimotor-linked reward settings. **(A–F)** The eye velocity as a function of time when the eye velocity in the previous trial was faster [**(A–C)** FE subgroup] or slower [**(D–F)** SE subgroup] than the average response of the base blocks. Solid lines represent eye responses on the current trial and dashed lines represent data on the prior one trial. The gray lines are base blocks and color lines are high block (red lines), low block (blue lines) and random block (orange lines). Cyan shading represents the steady state period. Error bars: Mean ± SEM. **(G–I)** Difference of mean trial-over-trial change in the steady state between the base block and its associated high **(G)**, low **(H)** or random **(I)** block. The FE (dark color) and SE (light color) subgroups are depicted, respectively. The data distributions of individual blocks were color-coded. Boxes indicate average values, with 25% quartile values and 75% quartile values. Error bars indicate minimum and maximum values across all blocks. ^**^p < 0.01; ^***^p < 0.001; Paired Student’s *t*-test.

We investigated how eye responses of the steady state of pursuit in the prior trial affected the ocular performances in the subsequent trial. The data were separated into the faster-experience (FE, Figures 6A–C) and slower-experience (SE, Figures 6D–F) subgroups based on whether the eye velocity during the steady-state phase in the previous trial was higher or lower than the threshold of eye responses in the base block. As shown by the example monkey’s eye traces in the base block, the faster eye movements during the steady state of pursuit in the prior trial are followed by slower eye velocities in the subsequent trial in the FE group (Figures 6A–C, black dashed lines vs. black solid lines), and in the SE group, the opposite is true (Figures 6D–F, black dashed lines vs. black solid lines). Relaxation of motor performance typically results in a return to the mean responses. When analyzing the data obtained from the low and random blocks (color lines in Figures 6B,C,E,F), it was observed that the size of the relaxation in the high block was significantly smaller in the FE group (red lines in Figure 6A, as determined by Bonferroni’s *post hoc* test, *p* < 0.05). Conversely, in the SE group, the size of the relaxation was larger (red lines in Figure 6D, as determined by Bonferroni’s *post hoc* test, *p* < 0.01). These findings were associated with an increase in eye velocities during the subsequent trial, as indicated by the red solid lines.

In order to assess mathematically the effect of motor experiences under distinct reward settings, we calculated the difference of trial-over-trial change in eye velocities during the steady state (cyan shading in Figure 6) between the base block and its corresponding high (Figure 6G), low (Figure 6H) or random (Figure 6I) block. Upon meticulous examination of the motor experiences observed in the previous trial, it was found that only rewarding greater eye responses can enhance eye movements on a single trial scale. This finding was supported by statistical analysis using a two-sided Student’s t-test, which revealed a significance level of *p* < 0.01 for all three monkeys in both the FE and SE groups (Figure 6G). In contrast, reward in the low or random block failed to modulate ocular responses significantly (Figures 6H,I, *p* > 0.05 for all three monkeys in both FE and SE groups). We further focused on the data in the high block. Even the eye velocities during the steady state in the FE group were substantially higher (1.7–2.7°/s) compared to the SE subgroup for all three monkeys (*p* < 0.001 for three monkeys). However, the enhancements by reward were not statistically significant for either faster or slower motor experiences. Therefore, it may be concluded that the facilitations of eye movements observed during the steady state cannot be attributed to motor experiences from the prior trial.

Next, we assessed whether the presence of a reward in the prior trial could enhance the ocular performances in the subsequent trial. The utilization of the random block for evaluation may be more advantageous due to the constraint imposed by the sensorimotor-linked reward rule in the high and low block, which limits the occurrence of reward and behavior. In the random block, equal chances of a reward in the proceeding trial were independent from prior motor performances. The delivery of reward in the prior trial, whether present or absent, did not result in significant changes in eye movement in the subsequent trial. It was determined by the use of a Paired Student’s *t*-test, with *p* > 0.16 observed for all three monkeys. Therefore, the monkeys’ pursuit eye movement during the steady state is not strongly influenced by the presence of a reward in a single trial.

## 4 Discussion

This study presents evidence that sensorimotor-linked rewards have a significant impact on behavioral performance, even in the absence of preceding sensory cues related to the forthcoming reward. The conclusions gained in the visual-guided pursuit task are derived from the effects resulting from altering the linkage between eye movements and reward. When the reward is associated with faster eye movements at the pursuit initiation, it facilitates pursuit velocity during the steady-state phase. In contrast, pursuit initiation is insensitive to the reward setting. Therefore, the modulation of sensorimotor-linked reward has a sensitive window of effect time. It functions on the motor-feedback-supported steady-state of pursuit, as opposed to the sensory-estimation-driven pursuit initiation. The facilitation of eye movements during steady-state pursuit significantly depended on the sensorimotor-linked reward setting, with a greater eye response linked with reward. Rewarding slower eye movements or randomizing the order of rewards did not effectively affect behavioral performance.

### 4.1 Sensorimotor-linked reward, a novel probe on reward interacting sensorimotor transformation

Previous research has revealed powerful effects of rewards through the utilization of sensory signals to inform forthcoming reward. A cued larger reward acted significantly to guide faster eye movements during both the initiation and the steady-state phases of pursuit to a single target motion in human (Brielmann and Spering, 2015). When monkeys were introduced to distinct combinations of reward sizes (small, medium, and large) in the two-target selection task, the pre-cued larger reward can bias smooth pursuit eye movement trajectories (Joshua and Lisberger, 2012). The bias led to a faster eye velocity in the direction of the highly rewarded target, starting from the initiation of pursuit. There were significantly faster eye movements when monkeys were given with rewards associated with large and medium sizes than medium and small sizes. The cued larger reward also leads to a larger expression of directional learning in pursuit, with a faster eye velocity in the learning direction starting from the onset of pursuit. Therefore, a larger reward indeed has a powerful modulation to facilitate the smooth pursuit eye movement. In these studies, the cue was introduced as early as the stage of motor preparation and directly informed the forthcoming reward. It is expected that the cue can elicit an anticipation of reward prior to the initiation of behavior. The anticipation could shape the neural network to potentiate sensorimotor transformation and drive vigorous behavioral performances.

In the sensorimotor-linked reward task of this study, reward was set in a given trial only when monkeys’ behavioral performances were satisfied with the requirement of the reward setting in that trial. The reward would be delivered or not depended on the sensorimotor performance in individual trials and dynamically changed across all trials. There was an absence of effect at the initiation of pursuit, which is in contrast to the significant effect observed in experiments that utilized cues. This suggests that the anticipation of reward was not modulated prior to motion onset in the sensorimotor-linked reward task. Thus, monkeys probably did not have a successful prediction of the forthcoming reward before the target motion starting, or even before the initiation of pursuit in our experiments.

Our results further demonstrated that the presence of a reward in prior trial did not yield a significant effect on pursuit eye movements during the steady state. The effect of sensorimotor-linked reward on the steady-state of pursuit required tens of trials’ experiences to be effective in high blocks. Therefore, it is evident that the observed effects of sensorimotor-linked reward can be interpreted as a result of the cognitive comprehension of the relationship between reward and behavioral performance, and/or the conditional connection between reward and the neuronal activities of the brain’s pursuit system (which will be further elaborated upon in the last section of the Discussion). By this design, the integration of sensorimotor-linked reward can serve as a dynamic testing platform for evaluating the intricate relationship between reward and sensorimotor transformation.

### 4.2 The modulation of pursuit during the steady state by sensorimotor-linked reward

**Figure 7:**
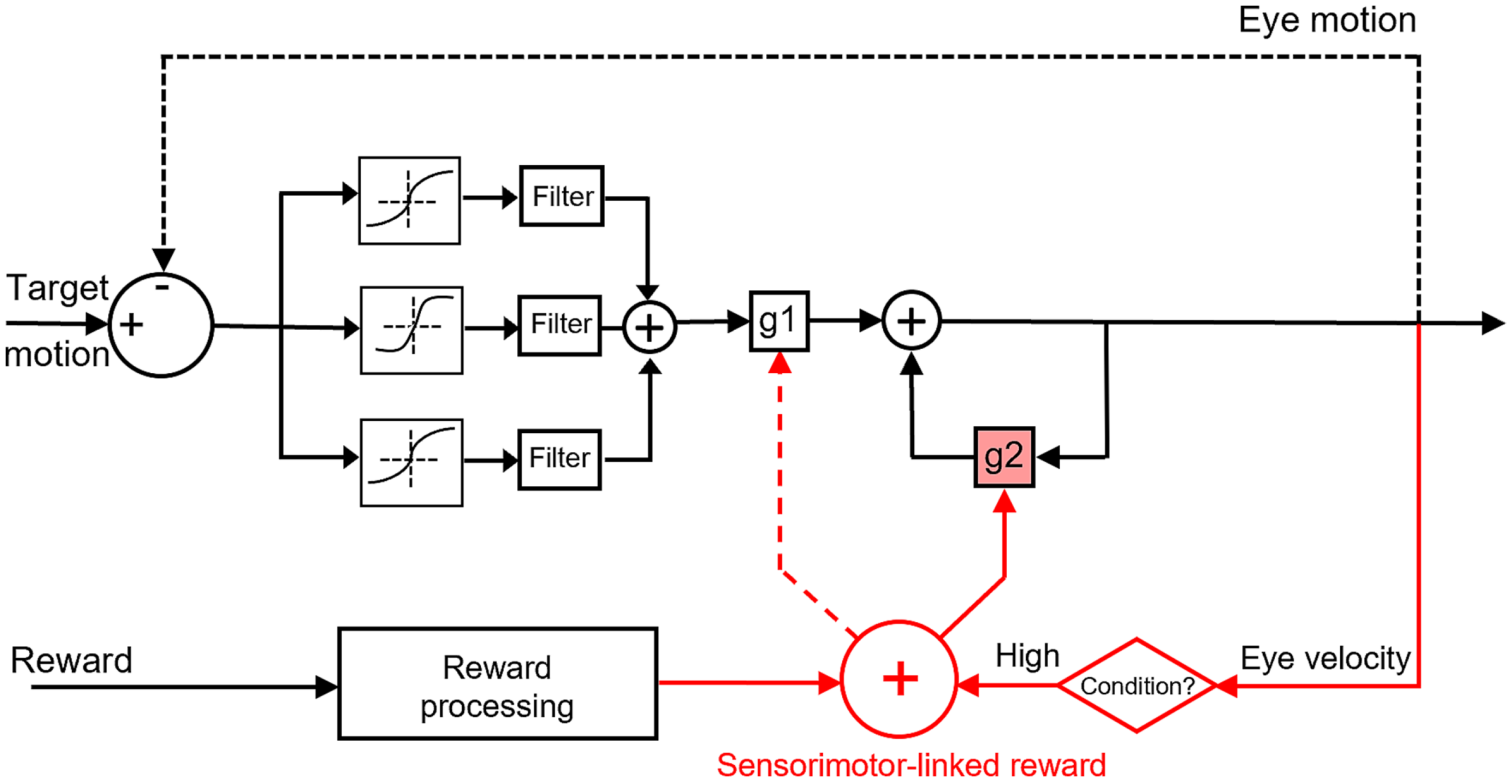
Conceptual scheme for sensorimotor-linked reward interacts with the sensorimotor transformation for smooth pursuit eye movements. Black schematic parts were inspired by Behling and Lisberger (2020). Red schematic parts were observed in this study. Our results depicted distinct reward interaction mechanisms for gain modulations in the pursuit system: the sensorimotor-linked reward can facilitate eye movements during the steady state (solid arrow to g2), as opposed to the initiation (dashed arrow to g1). Rewarding greater eye movements could work as a condition between the motor feedback and reward processing to elicit reward modulation on the neural system.

Pursuits, as ocular responses, encompass continuous eye movements to smoothly track the motion of attentional objects, with the initiation and the subsequent steady state. The initiation of pursuit refers to the initial response of the visual motion pathways towards a moving target, typically with a duration of about 100 ms. The transmission of visual signals through the visual system has not yet elapsed sufficiently to rectify the ongoing pursuit eye velocity during the initiation period. The duration of the second steady state extends from the commencement of the initiation until the cessation of the tracking. This period is distinguished by the utilization of online adjustments in pursuit eye velocity to counteract the occurrence of retinal slip. Behling and Lisberger recently studied the initiation and steady state of smooth pursuit eye movements by exploiting low-coherence patches of dots (Behling and Lisberger, 2020). They discovered that the reliability of visual motion signals can independently modify these two phases of pursuit. Reduction in the dot coherence decreases eye velocity during both periods, with quantitative distinctions between the sensory-driven initiation of pursuit and the motor-supported steady-state of pursuit. Their results indicate there are separate modulation of visual information on the strength of visual-motor transmission and motor feedbacks, distinct mechanisms on these two phases (gain terms: g1 and g2 in Figure 7).

Our study was designed to investigate how smooth pursuit eye movements were affected by the association of sensorimotor performance and reward. The results indicated that the manipulation of sensorimotor-linked reward has a significant impact on eye movements during the steady state, as opposed to the initiation, under a specific reward setting. The reward associated with faster eye movements during the initiation enhances pursuit velocity during the steady state. In contrast, the pursuit initiation demonstrated an inability to induce changes in latency, eye velocity, and their variances when tracking a moving target, regardless of the reward settings. The findings of this study revealed that eye movements’ facilitation during steady-state pursuit was significantly dependent on the sensorimotor-linked reward setting, with a greater ocular response linked to reward. Neither rewarding slower eye movements or in a randomized order did not substantially affect behavioral performance. Based on these observed behavioral outcomes, a conceptual scheme for sensorimotor-linked reward interacts with the sensorimotor transformation for smooth pursuit eye movements was depicted in Figure 7. Eye movements during the initiation of pursuit might serve as a condition to trigger reward modulation on the neural system in this study. The sensorimotor-linked reward can effectively modulate the steady state of pursuit (g2) via the conditioner, while it may not be insensitive to the initiation (g1).

The underlying reason for the absence of reward effects on the initiation in the study remains an intriguing question. The initiation phase of pursuit is a pre-attentive stage driven by retinal slip signals, and it appears to be unaffected by top-down attention or other cognitive functions (Souto and Kerzel, 2021). Previous studies have demonstrated that naïve monkeys can significantly improve their ocular tracking movements over the course of several weeks of training (Bourrelly et al., 2016; Hietanen et al., 2017; Botschko et al., 2018). In this study, monkeys underwent rigorous training for a couple of months before data collection, and exhibited proficient capabilities in initiating eye movements. These factors could hinder the modulation of eye responses during the initiation phase by the voluntary efforts related to the reward. During the steady-state phase of pursuit, eye movements are primarily guided by real-time feedback signals, even in monkeys who have undergone extensive training. In contrast to the initiation of pursuit, the steady-state pursuit requires attentional tracking (Souto and Kerzel, 2021). Hence, the neural system that maintains eye movements during the steady-state phase may have higher flexibility towards the sensorimotor-linked reward, compared to the system engaged in initiating pursuit during the initiation. These factors could contribute to the distinct regulation of eye movements during the initiation and steady state of pursuit. However, further research is needed to verify their significance.

### 4.3 Possible sites of the sensorimotor-linked reward modulation on pursuit

Smooth pursuit eye movements are mediated by a cerebro-ponto-cerebellar pathway, with distinct neural mechanisms for the initiation and steady state of pursuit. Neurons in the striate and extrastriate visual cortex provide sensory information that accurately drives the initiation of pursuit (Newsome et al., 1985; Priebe et al., 2003). Based on the population responses of neurons in the middle temporal area (MT) and/or the middle superior temporal visual area (MST), the pursuit system estimates the target’s speed and direction. The majority of the observed variations in eye velocity during the initiation period can be attributed to sensory estimation errors (Osborne et al., 2005), which are closely correlated with variations in neural activity in area MT (Hohl et al., 2013; Lee and Lisberger, 2013; Lee et al., 2016). In contrast, MT neurons exhibit a lack of responsiveness when they come to guide the steady-state tracking (Maunsell and Vanessen, 1983; Morris and Lisberger, 1987; Behling and Lisberger, 2020). The ability to maintain eye velocity during the steady state is predominantly accomplished through the utilization of an “extraretinal signal.” This signal could be generated by previous eye movement commands and is believed to be associated with the positive feedback mechanism of eye velocity corollary discharge (Churchland and Lisberger, 2001; Churchland et al., 2003; Krauzlis, 2003). Several prior studies have been undertaken to investigate the role of the frontal eye fields (FEF) and MST in pursuit (Gottlieb et al., 1994; Tian and Lynch, 1996; Tanaka and Lisberger, 2001; Ono, 2015). Neurons in FEF and MST continue to discharge during the steady state of pursuit, even though image motion in the retina is small or zero. Both of them appear to transmit extraretinal signals related to the ongoing eye movement to the cerebellum. Recordings of Floccular Purkinje cells indicate that the cerebellum receives sensory input via the sensory route, as well as positive feedback about motor commands (Miles and Fuller, 1975; Lisberger and Fuchs, 1978; Shidara et al., 1993). These inputs are then utilized by the cerebellum to generate simple-spike firing patterns that represent a kinematic model of eye movements (Shidara et al., 1993; Medina and Lisberger, 2009). The studies discussed above suggest that the key nodes of the pursuit system, such as the cerebellum, FEF and MST, may be responsible for transmitting the “extraretinal signal” involved in maintaining steady-state pursuit (Lisberger, 2015).

Reward-related signals are present in a wide range of brain subareas. What is the nature of the interaction between reward and the sensorimotor transformation in the pursuit system, and where does this interaction occur? The role of area FEF has been extensively studied in relation to its contribution to cue-guided reward processing (Roesch and Olson, 2003, 2005; Glaser et al., 2016; Lixenberg and Joshua, 2018). Recently, rising evidence demonstrated that the cerebellum encodes the expectation of reward in both cerebellar granule cells (Wagner et al., 2017) and cerebellar Purkinje cells’ complex-spike firing (Kostadinov et al., 2019; Larry et al., 2019; Sendhilnathan et al., 2021). Meanwhile, the cue-guided reward information is integrated into cerebellar simple-spike activities, which could guide behavioral responses (Lixenberg et al., 2020; Sendhilnathan et al., 2020). Researches on the cerebellar plasticity of motor learning in pursuit further suggested that the cerebellum may have the capacity to receive both motor feedback and reward information (Yang and Lisberger, 2010; Heffley et al., 2018).

Posed in this manner, the incorporation of reward-related information has the potential to interact with the pursuit system through integrating signals into the cerebellum and FEF. These crucial nodes in the neural circuit for pursuit could work as conductors facilitated by the feedback of “extraretinal signals” to maintain the pursuit steady state. The findings of our study indicate that the “extraretinal signals” may encompass the reward associated with sensorimotor performances. There was a lack of neurophysiological data to verify the hypothesis in this behavioral study. The manner in which reward information is represented in the pursuit system (FEF and cerebellum) offers a potential explanation for why the reward substantially modified smooth pursuit eye movements during the steady state, as opposed to the initiation period (solid arrow to g2 vs. dashed arrow to g1, Figure 7). This is also consistent with the observed behavioral results in this study.

During our investigations, the monkeys consistently encountered trials that involved the presence of a reward, as well as trials that did not offer any reward, under distinct sensorimotor-linked reward settings. Smooth pursuit eye movements are selectively sensitive to the reward setting associated with behavioral performances. Hence, there exists a challenge in current study to comprehensively understand the underlying reasons behind the exclusive reliance on rewarding greater eye movements during the initiation of pursuit. The observation implies that the neuronal nodes within the pursuit system may also contribute to the processing of rewards and remodel neural coding to lead smooth pursuit eye movements. Future research should address these limitations and questions to examine how the association between reward and greater eye responses serves as a condition to elicit reward modulation on the neural system. It is imperative to conduct following research on this matter in the near future.

## Data availability statement

The original contributions presented in the study are included in the article/supplementary material, further inquiries can be directed to the corresponding author.

## Ethics statement

The animal study was approved by the Institutional Animal Administration Committees at the Institute of Biophysics, Chinese Academy of Sciences. The study was conducted in accordance with the local legislation and institutional requirements.

## Author contributions

YH: Data curation, Formal analysis, Investigation, Validation, Visualization, Writing – original draft, Writing – review & editing. HW: Formal analysis, Funding acquisition, Investigation, Writing – review & editing. MJ: Methodology, Writing – review & editing. YY: Conceptualization, Formal analysis, Funding acquisition, Investigation, Methodology, Project administration, Supervision, Visualization, Writing – original draft, Writing – review & editing.
